# ﻿*Cremastobombyciasocoromaensis* sp. nov., the first South American representative of the micromoth genus *Cremastobombycia* Braun (Lepidoptera, Gracillariidae, Lithocolletinae)

**DOI:** 10.3897/zookeys.1218.135606

**Published:** 2024-11-25

**Authors:** Héctor A. Vargas

**Affiliations:** 1 Departamento de Recursos Ambientales, Facultad de Ciencias Agronómicas, Universidad de Tarapacá, Arica, Chile Universidad de Tarapacá Arica Chile

**Keywords:** Andes, Chile, leaf miner, new record, new species, taxonomy

## Abstract

The micromoth *Cremastobombyciasocoromaensis***sp. nov.** (Lepidoptera, Gracillariidae, Lithocolletinae) from the arid highlands of the western slope of the Andes of northern Chile is described and illustrated. Larvae construct bulged leaf mines on the shrub *Baccharistola* Phil. (Asteraceae). Pupation occurs in a silk cocoon constructed by the last instar larva inside the mine. The cocoon and the mine surface are pierced by the frontal process of the pupa to allow adult emergence. This discovery represents the first record of *Cremastobombycia* Braun, 1908 in South America.

## ﻿Introduction

*Cremastobombycia* Braun, 1908 (Lepidoptera, Gracillariidae, Lithocolletinae) was originally described as a subgenus of *Lithocolletis* Hübner, 1825, currently a synonym of *Phyllonorycter* Hübner, 1823 (De Prins and De Prins 2024), to include five North American micromoth species bearing forewing and hindwing with veins M1 and M2 stalked and labial palpus with the third palpomere slightly longer than the second one (Braun 1908). Furthermore, Braun (1908) indicated some elements of the wing pattern, larval morphology, and mine and cocoon shape shared by these five species. Busck (1910) treated *Cremastobombycia* as a genus, and Meyrick (1912) subsequently designated *Lithocolletissolidaginis* Frey & Boll, 1876 as its type species. Vári (1961) provided a more detailed description based on the study of the adult stage of the type species, including genitalia morphology. De Prins and Kawahara (2012) expanded this definition to include variations in forewing venation and genitalia. Finally, Davis et al. (2013) highlighted a morphological specialization of the hypopharynx of the larva as a distinctive character. Consistent with the evolutionary closeness between *Cremastobombycia* and *Phyllonorycter* suggested early based on morphology (Braun 1908, 1909; Busck 1910), results of molecular analyses have provided support for a sister relationship between these two genera (Kawahara et al. 2011, 2017; De Prins and Kawahara 2012).

Shortly after the original description of *Cremastobombycia*, an additional species was described based on the type material collected in Honolulu, Oahu (Busck 1910). However, this species had previously been purposely introduced to the Hawaiian Islands from Mexico, which represents its native range, as a biocontrol for *Lantanacamara* L. (Verbenaceae) (Busck 1910). Thus, after this addition, the genus continued to include species exclusively native to North America, a picture that remained unchanged for almost 100 years, until the recent discovery of two African representatives from Kenya and Tanzania (De Prins and Kawahara 2012). The subsequent discovery of another North American member from Florida (Davis et al. 2013) brought the currently described species of *Cremastobombycia* to nine (De Prins and De Prins 2024). However, this number should continue to grow in the near future, as Davis et al. (2013) reported at least seven other undescribed species from North America.

*Cremastobombycia* larvae are leaf miners whose feeding activity produces longitudinally very wrinkled mines mainly found on the underside of the leaf (Braun 1908), known as tentiform mines (Davis et al 2013). As in many Gracillariidae lineages, the hypermetamorphic development of *Cremastobombycia* larvae includes two distinct forms: an early sap-feeding form with prognathous mouthparts and flattened thorax and abdomen, and a later tissue-feeding form with hypognathous mouthparts and cylindrical thorax and abdomen (Davis et al. 2013). Host plants have been recorded for the seven North American species (Braun 1908; Busck 1910; Davis et al. 2013; De Prins and De Prins 2024), while those of the two African species remain unknown (De Prins and Kawahara 2012). The available records suggest a main association with Asteraceae, as six species are hosted by plants of this family and only one by members of Verbenaceae (Braun 1908; Busck 1910; Davis et al. 2013; De Prins and De Prins 2024). In contrast, only two species of the highly diverse *Phyllonorycter* are associated with members of Asteraceae (Vári 1961, De Prins and Kawahara 2012).

No *Cremastobombycia* species have been reported from South America (De Prins et al. 2019). However, morphological examination revealed that micromoths obtained from leaf mines collected in the arid highlands of the Andes of northern Chile belong to an undescribed species of this genus. The aim of this contribution is to provide a formal taxonomic description for the first South American member of *Cremastobombycia*.

## ﻿Materials and methods

Mined leaves of the shrub *Baccharistola* Phil. (Asteraceae) were collected in May 2023 in the surroundings of Socoroma Village (18°17'22"S, 69°35'12"W) at about 3400 m elevation on the western slope of the Andes in the Parinacota Province of northern Chile. Adults emerged in June 2023. The abdomen of each adult was removed and placed in hot KOH 10% for a few minutes for dissection of the genitalia, which were stained with Eosin Y and Chlorazol Black and mounted on slides with Euparal. Photos of the adults were taken with an iPhone 11 camera attached to a Leica M125 stereomicroscope. Photos of the genitalia were taken with a Leica MC170 HD digital camera attached to a Leica DM1000 LED light microscope. The holotype, paratypes and their genitalia slides are deposited in the “Colección Entomológica de la Universidad de Tarapacá” (IDEA), Arica, Chile.

## ﻿Results

### ﻿ Cremastobombycia socoromaensis sp. nov.

Taxon classificationAnimaliaLepidopteraGracillariidae

5C10BC46-AB8B-59CF-AB9E-AC3291CF0093

https://zoobank.org/6E904F72-B578-4B30-8CD2-B6F7A8516AAE

Figs 1 , 2 , 3 , 4

#### Type locality.

Chile, Parinacota Province, Socoroma (18°17'22"S, 69°35'12"W), 3400 m elevation on the western slope of the Andes.

#### Type material.

***Holotype*.** Chile • ♂; Parinacota, Socoroma; June, 2023; H.A. Vargas leg.; ex-larva; *Baccharistola*; May, 2023; “HOLOTYPE *Cremastobombyciasocoromaensis* Vargas” [red handwritten label]; IDEA-LEPI-2024-09; HAV-1811 [genitalia slide] (IDEA). ***Paratypes*.** Chile • 2♂ 2♀; same data as for the holotype; IDEA-LEPI-2024-10 to IDEA-LEPI-2024-13; HAV-1639, 1719, 1806, 1807 [genitalia slides] (IDEA).

#### Diagnosis.

Among the currently described *Cremastobombycia* species, *C.socoromaensis* sp. nov. is recognized based on wing pattern and genitalia morphology. Male forewing (length 3.6–3.7 mm) is brownish-orange and bears poorly defined creamy-white markings: a short longitudinal sub-basal streak and three costal and two dorsal oblique strigulae. Although female forewing (length 3.0–3.1 mm) is also brownish-orange with a sub-basal streak similar to that of the male, the four well-defined creamy-white transverse fasciae and three well-defined dark brown spots differ from the forewing pattern of the male. Male genitalia of *C.socoromaensis* sp. nov. resemble those of *C.chromolaenae* Davis, 2013 from Florida, United States. However, the poorly defined longitudinal sub-basal streak on the forewing of the former clearly contrasts with the conspicuous white longitudinal streak along the basal third on the forewing of the latter (Davis et al. 2013, figs 2, 3). Furthermore, the posterior projection of the tegumen, the straight margin between the lobes of the transtilla, the vesica with a cornutus in the male genitalia, and the diamond-shaped signum with a transverse fold in the female genitalia allow the recognition of *C.socoromaensis* sp. nov.; as in the male genitalia of *C.chromolaenae* the tegumen lacks a posterior projection, the margin between the lobes of the transtilla is concave, and the vesica lacks a cornutus (Davis et al. 2013, figs 6, 8), and the female genitalia have a strongly bilobed signum (Davis et al. 2013, figs 9–12). The transverse fold of the signum in the female genitalia of *C.socoromaensis* sp. nov. resembles that of *C.lantanella* (De Prins et al. 2019, fig. 436). However, in clear contrast with the male genitalia of the former, those of *C.lantanella* lack a posterior projection of the tegumen, have the saccus shorter than the vinculum width, and lack a cornutus on the vesica (De Prins et al. 2019, fig. 374).

#### Description.

**Figure 1.**
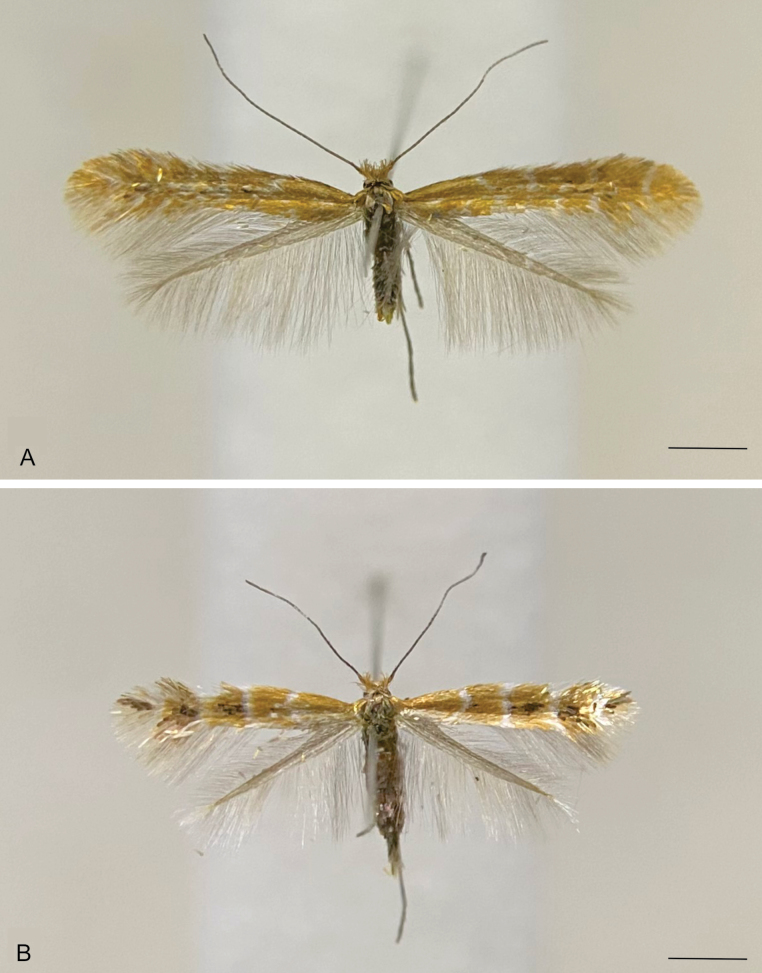
Habitus of *Cremastobombyciasocoromaensis* sp. nov. (Lepidoptera, Gracillariidae) **A** holotype, male **B** paratype, female. Scale bars: 1 mm.

**Figure 2.**
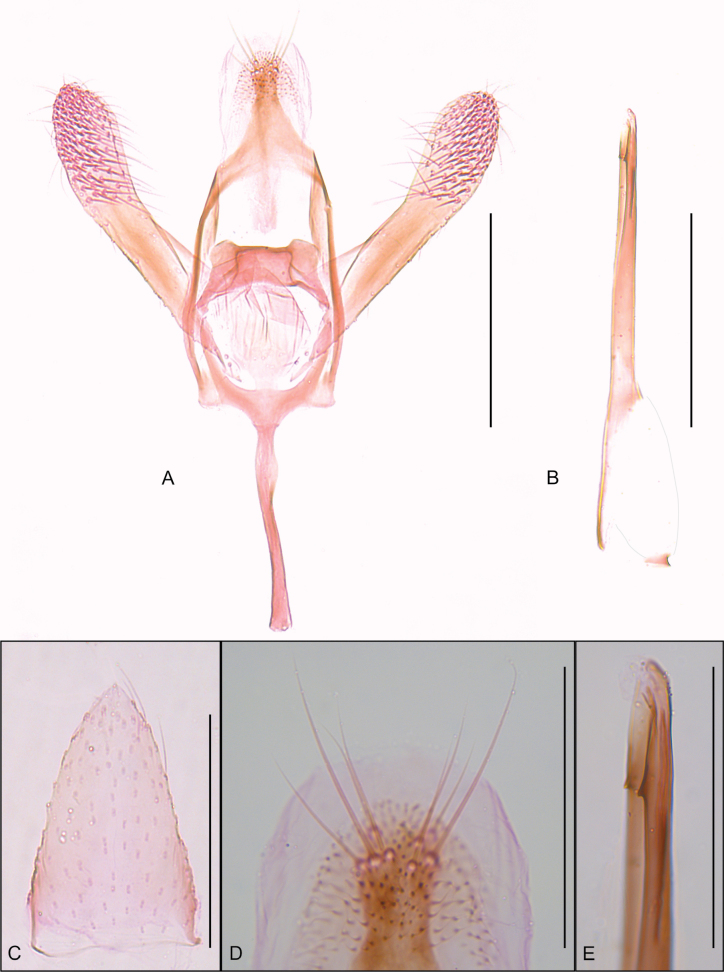
Male genitalia and sternum VIII of *Cremastobombyciasocoromaensis* sp. nov. (Lepidoptera, Gracillariidae) **A** male genitalia, phallus removed **B** phallus **C** sternum VIII **D** tegumen apex **E** phallus apex. Scale bars: 0.2 mm (**A, B**); 0.1 mm (**C–E**).

**Male** (Fig. 1A). ***Head*.** Vertex with narrow, elongate, raised scales, mostly brownish-orange and a few dark brown; frons with narrow, elongate, smooth brownish-orange scales. Antenna filiform, slightly shorter than forewing, silvery-gray, scape with pecten. Labial palpus straight, drooping, silvery-gray. ***Thorax*** (forewing length 3.6–3.7 mm). Mostly brownish-orange with scattered creamy-white dorsally; silvery-gray ventrally; legs silvery-gray. Forewing brownish-orange with poorly defined creamy-white markings, including a short longitudinal sub-basal streak and three costal and two dorsal oblique strigulae; first two costal strigulae arising before the middle, third one arising near the apex; dorsal strigulae arising near the middle; scattered dark brown scales between the two dorsal strigulae and between the second dorsal and the third costal strigulae; fringe brownish-orange. Hindwing uniformly gray with gray fringe. ***Abdomen*.** Mostly gray with scattered creamy-white scales; sternum VIII flap-like, elongate. ***Male genitalia*** (Fig. 2). Tegumen with narrow arms slightly widened on dorsal half, with flat, somewhat triangular posterior projection bearing eight elongate setae near apex. Vinculum U-shaped. Saccus a narrow, elongate, slightly sinuous rod. Subscaphium a narrow, poorly sclerotized longitudinal stripe ending in a broad patch of microtrichiae. Juxta a broad, poorly sclerotized plate joined to posterior margin of vinculum by a narrow stripe. Transtilla well-differentiated with two widely separated lobes on anterior margin. Valva elongate, slender, length about 1.5 times the saccus; dorsal margin slightly convex near tip; ventral margin mostly straight; apex widely rounded; median surface with dense patch of stout setae on distal third. Phallus cylindrical, straight, about twice the saccus length; coecum about a third the phallus length; a narrow cleft on distal third with two small spine-like projections on opposed margins; vesica with a narrow, elongate cornutus; ductus ejaculatorius with a ring-shaped sclerite near the tip of the coecum.

**Figure 3.**
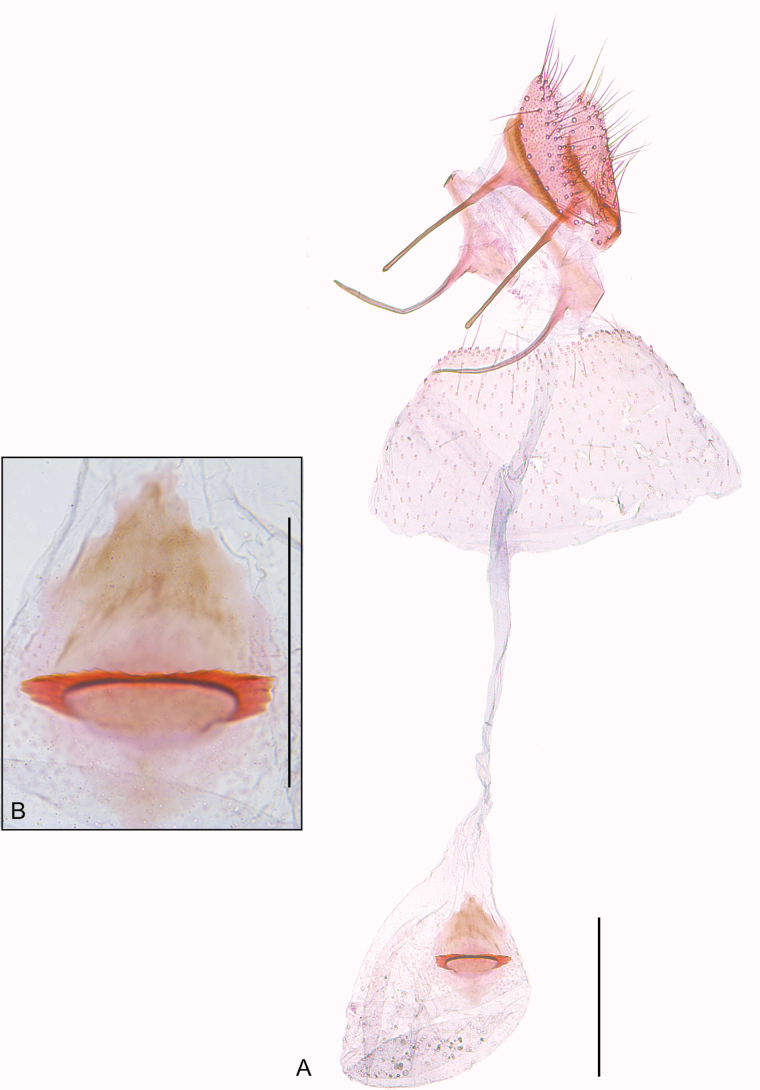
Female genitalia of *Cremastobombyciasocoromaensis* sp. nov. (Lepidoptera, Gracillariidae) **A** female genitalia **B** signum. Scale bars: 0.2 mm (**A**); 0.1 mm (**B**).

**Female** (Fig. 1B). Mostly similar to male, except for forewing length (3.0–3.1 mm) and maculation pattern; mostly brownish-orange with a poorly defined, short creamy-white longitudinal sub-basal streak and four well-defined creamy-white transverse fasciae, the first one convex, the three other straight; a small dark brown spot on the middle of the outer margin of the first fascia; a great dark brown spot between the outer margin of the second and the inner margin of the third fasciae and between the outer margin of the third and the inner margin of the fourth fasciae; a small dark brown spot on the outer margin of the fourth fascia. ***Female genitalia*** (Fig. 3). Papillae anales flattened, bearing long setae mostly near posterior margin. Posterior apophyses straight, slightly longer than posterior margin of papillae anales. Anterior apophyses dorsally curved, length similar to posterior apophyses. Ostium near the posterior margin of sternum VII. Ductus bursae membranous, narrow, about three times the posterior apophyses length; ductus seminalis arising near the posterior third of ductus bursae. Corpus bursae membranous, oval, about half the length of ductus bursae; signum a slightly sclerotized diamond-shaped plate on posterior half of corpus bursae with a well-sclerotized semicircular serrated transverse fold near the middle.

#### Etymology.

The specific epithet is derived from the type locality.

#### Distribution

**Figure 4.**
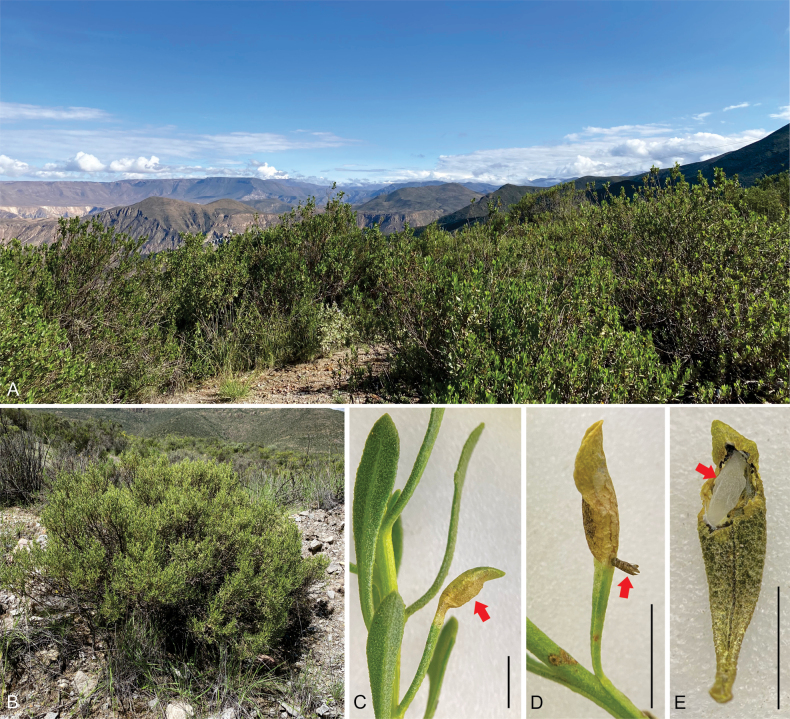
Natural history of *Cremastobombyciasocoromaensis* sp. nov. (Lepidoptera, Gracillariidae) **A** habitat at the type locality **B** host plant *Baccharistola* (Asteraceae) **C** leaf mine (red arrow) on *B.tola***D** pupal exuvium (red arrow) attached to the mine after adult emergence **E** artificially opened leaf mine showing a pupal cocoon (red arrow). Scale bars: 5 mm.

**(Fig. 4A).** The currently documented range of *C.socoromaensis* sp. nov. is restricted to the type locality in the surroundings of Socoroma Village, at about 3400 m elevation on the western slope of the Andes in the Parinacota Province of northern Chile.

#### Host plant

**(Fig. 4B).***Baccharistola* is the only host plant currently recorded for *C.socoromaensis* sp. nov. This shrub, native to Argentina, Bolivia, Chile and Peru (POWO 2024), is used for medicinal purposes (Villagrán et al. 2003). In northern Chile, *B.tola* inhabits the highlands of the Andes between about 2000–4800 m elevation (Rodriguez et al. 2018).

#### Natural history

**(Fig. 4C–E).** Eggs of *C.socoromaensis* sp. nov. are laid individually mainly on the adaxial surface of the leaf. Larva and pupa are endophytic. The hypermetamorphic larval development includes early sap-feeding and later tissue-feeding forms. The bulged, elongate mature mine occupies a large proportion of the leaf. The last instar tissue-feeding larva constructs a loose, smooth, cylindrical silk cocoon for pupation attached to the mine by anterior and posterior ends. The cocoon and the mine surface are pierced by the frontal process of the pupa to allow the adult emergence.

## ﻿Discussion

Besides the morphological attributes of the labial palpus and wing venation of the adult stage highlighted in the original description of *Cremastobombycia*, Braun (1908) indicated that members of this genus use host plants of the family Asteraceae, construct longitudinally very wrinkled (=tentiform) mines on the lower side of the leaf, and pupate in a dense, elongate cocoon suspended inside the mine by silken threads at the anterior and posterior ends. Although these features are found in many of the North American species of *Cremastobombycia*, mines of *C.grindeliella* (Walsingham, 1891) sometimes occur on the upper side of the leaf (Braun 1908), and the hosts of *C.lantanella* belong to the family Verbenaceae (Busck 1910). Meanwhile, host plants, leaf mines and immature stages of the African species remain unknown (De Prins and Kawahara 2012).

The inclusion of *C.socoromaensis* sp. nov. in *Cremastobombycia* is based on its wing venation, which is identical to that of the type species (Meyrick 1912; Vári 1961; Davis et al. 2013), and its genitalia morphology, which fits the pattern previously described for this genus (Vári 1961; De Prins and Kawahara 2012; Davis et al. 2013). Furthermore, *B.tola* belongs to the family most frequently reported as host to members of *Cremastobombycia* (De Prins and De Prins 2024). Although preliminary observations using light microscopy suggest that the last instar tissue-feeding larva of *C.socoromaensis* sp. nov. bears six lobes like those indicated by Davis et al. (2013) as a distinctive feature of the genus, further studies using scanning electron microscopy will be needed to perform detailed observations of these small structures.

*Cremastobombyciasocoromaensis* sp. nov. is the first species of the genus described from South America. Like other recent studies (Cerdeña et al. 2020, 2022; Vargas-Ortiz et al. 2020), this discovery highlights the need to continue the search for leaf miners associated with plants native to the western slope of the central Andes to improve the understanding of the diversity of Gracillariidae which remains overlooked in these arid environments.

## Supplementary Material

XML Treatment for Cremastobombycia socoromaensis

## ﻿Additional information

### Author ORCIDs

Héctor A. Vargas https://orcid.org/0000-0002-5355-3157

### Data availability

All of the data that support the findings of this study are available in the main text.

## References

[B1] Braun A (1908) Revision of the North American species of the genus *Lithocolletis* Hübner. Transactions of the American Entomological Society 34: 269–357. 10.5962/bhl.title.17825

[B2] Braun AF (1909) Phylogeny of the Lithocolletid group. (Preliminary Survey.). Canadian Entomologist 41: 419–423. 10.4039/Ent41419-12

[B3] Busck A (1910) New Central-American Microlepidoptera introduced into the Hawaiian Islands. Proceedings of the Entomological Society of Washington 12: 132–135.

[B4] Cerdeña J Farfán J Vargas HA Brito R Gonçalves GL Lazo A Moreira GRP (2020) *Phyllocnistisfurcata* sp. nov.: A new species of leaf-miner associated with *Baccharis* (Asteraceae) from Southern Peru (Lepidoptera, Gracillariidae). ZooKeys 996: 121–145. 10.3897/zookeys.996.53958 33312049 PMC7710689

[B5] Cerdeña J Farfán J Vargas HA Huanca-Mamani W Gonçalves GL Moreira GRP (2022) A contribution to the knowledge of leaf-mining *Phyllocnistis* Zeller, 1848 associated with *Baccharis* (Asteraceae), with description of two new species from Peru (Lepidoptera: Gracillariidae). Zootaxa 5104: 196–208. 10.11646/zootaxa.5104.2.2 35391041

[B6] Davis DR Diaz R Overholt WA (2013) Systematics and biology of *Cremastobombyciachromolaenae*, new species (Gracillariidae), a natural enemy of *Chromolaenaodorata* (L.) King and H. Robinson (Asteraceae). Journal of the Lepidopterists Society 67: 35–41. 10.18473/lepi.v67i1.a4

[B7] De Prins J De Prins W (2024) Global Taxonomic Database of Gracillariidae (Lepidoptera). http://www.gracillariidae.net [accessed 05 August 2024]

[B8] De Prins J Kawahara AY (2012) Systematics, revisionary taxonomy, and biodiversity of Afrotropical Lithocolletinae (Lepidoptera: Gracillariidae). Zootaxa 3594: 1–283. 10.11646/zootaxa.3594.1.1

[B9] De Prins J Arévalo-Maldonado H Davis DR Landry B Vargas HA Davis MM Brito R Fochezato J Oshima I Moreira GRP (2019) An illustrated catalogue of the Neotropical Gracillariidae (Lepidoptera) with new data on primary types. Zootaxa 4575: 1–110. 10.11646/zootaxa.4575.1.1 31715785

[B10] Kawahara AY Ohshima I Kawakita A Regier JC Mitter C Cummings MP Davis DR Wagner DL De Prins J Lopez-Vaamonde C (2011) Increased gene sampling strengthens support for higher-level groups within leaf-mining moths and relatives (Lepidoptera: Gracillariidae). BMC Evolutionary Biology 11: 182. 10.1186/1471-2148-11-182 PMC3145599 21702958

[B11] Kawahara AY Plotkin D Ohshima I Lopez-Vaamonde C Houlihan PR Breinholt JW Kawakita A Xiao L Regier JC Davis DR Kumata T Sohn J-C De Prins J Mitter C (2017) A molecular phylogeny and revised higher-level classification for the leaf-mining moth family Gracillariidae and its implications for larval host-use evolution. Systematic Entomology 42: 60–81. 10.1111/syen.12210

[B12] Meyrick E (1912) LepidopteraHeterocera (Tineae). Fam. Gracilariadae. In: Wytsman P (Ed.) Genera Insectorum.Fascicule 128. V. Verteneuil & L. Desmet, Imprimeurs-Éditeurs, Bruxelles, 1–36.

[B13] POWO (2024) Plants of the World Online. Royal Botanic Gardens, Kew. https://powo.science.kew.org/ [Retrieved 21 August 2024]

[B14] Rodriguez R Marticorena C Alarcón D Baeza C Cavieres L Finot VL Fuentes N Kiessling A Mihoc M Pauchard A Ruiz E Sanchez P Marticorena A (2018) Catálogo de las plantas vasculares de Chile. Gayana. Botánica 75: 1–430. 10.4067/S0717-66432018000100001

[B15] Vargas-Ortiz M Aliaga-Pichihua G Lazo-Rivera A Cerdeña J Farfán J Huanca-Mamani W Vargas HA (2020) Cryptic Diversity in the Monotypic Neotropical Micromoth Genus *Angelabella* (Lepidoptera: Gracillariidae) in the Peru-Chile Desert. Insects 11: 677. 10.3390/insects11100677 PMC7601689 33036122

[B16] Vári L (1961) South African Lepidoptera. Vol. I. Lithocolletidae. Transvaal Museum Memoir 12: 1–238.

[B17] Villagrán C Romo M Castro V (2003) Etnobotánica del sur de los Andes de la Primera Región de Chile: Un enlace entre las culturas altiplánicas y las de quebradas altas del Loa superior. Chungara (Arica) 35: 73–124. 10.4067/S0717-73562003000100005

